# Identification of SNPs in the *NKA* Gene of *Scylla paramamosain* and the Association Analysis with Low-Salinity Tolerance

**DOI:** 10.3390/ijms27020920

**Published:** 2026-01-16

**Authors:** Chunyan Yin, Zhiqiang Liu, Keyi Ma, Wei Wang, Lingxiao Wang, Lingbo Ma, Chunyan Ma, Fengying Zhang

**Affiliations:** 1 Key Laboratory of East China Fishery Resources Exploitation, Ministry of Agriculture and Rural Affairs, East China Sea Fisheries Research Institute, Chinese Academy of Fishery Science, Shanghai 200090, China; 2 College of Fisheries and Life Science, Shanghai Ocean University, Shanghai 201306, China

**Keywords:** Na^+^/K^+^-ATPase, SNPs, low salinity, *Scylla paramamosain*, expression, association

## Abstract

The Na^+^/K^+^-ATPase (NKA) gene encodes a critical membrane transporter that maintains cellular ion homeostasis and plays a pivotal role in osmoregulation and salinity adaptation of aquatic organisms. In this study, we identified and validated SNP markers in the NKA gene associated with low-salinity tolerance in *Scylla paramamosain*. Four candidate SNPs (g.72037G>T, g.72122G>C, g.74293G>T, and g.74433G>T) were screened and genotyped in low-salinity tolerant and intolerant groups. Association analysis revealed that mutant genotypes at all four loci were significantly enriched in the tolerant group (*p* < 0.05), with odds ratios (OR) > 1. The tolerant group exhibited higher genetic diversity parameters than the intolerant group. Haplotype analysis showed the GGGG haplotype was dominant in the intolerant group, whereas the other haplotypes were mainly enriched in the tolerant group. The NKA expression in the mutant genotypes was significantly higher than that in the wild genotypes by qRT-PCR. For tolerant individuals, the fast-growing group exhibited higher mutation frequencies than the slow-growing group. Multi-locus analysis achieved substantially more discrimination accuracy than single-locus analysis. These findings demonstrated that these SNPs could be candidate molecular markers for breeding programs in *S. paramamosain* in low-salinity environments, helping to identify individuals with enhanced salinity tolerance and supporting sustainable aquaculture practices.

## 1. Introduction

Salinity is a critical environmental factor in aquatic ecosystems, which profoundly influences the physiological performance, growth, and survival of aquatic crustaceans [1,2]. Na^+^/K^+^-ATPase (NKA) functions as an important transmembrane ion transporter and plays a central role in salinity adaptation. It actively maintains the electrochemical gradients of intracellular and extracellular Na^+^ and K^+^ through ATP hydrolysis to drive multiple secondary active transport processes [3]. In crustaceans, the activity and expression of NKA exhibit salinity-dependent and tissue-specific patterns due to salinity reduction [4,5,6,7,8,9]. However, the regulation of *NKA* under low-salinity stress was involved in multiple pathways. Under low-salinity conditions, the biological processes are rapidly activated [10]. At the transcriptional level, these pathways coordinate or complement the regulatory patterns of *NKA* and multiple ion transport genes such as *CLC*, *CCP*, *NKCC*, and *NHE*; this coordination modulates transport efficiency via ion gradient coupling and collectively maintains ionic homeostasis under low-salinity stress, ultimately forming a multilayered osmoregulatory network centered on *NKA* [10,11,12].

Single-nucleotide polymorphisms (SNPs) have many advantages, such as wide distribution, high density, and correlation with traits [13,14]. Characterization analysis of the predicted SNPs in *Litopenaeus vannamei* showed that the estimated SNP frequency was 0.21% (one SNP per 476 bp) [15], while in *Macrobrachium nipponense*, the average density of SNPs ranged from 1.356 SNPs/1000 kb (Chr43) to 4.349 SNPs/1000 kb (Chr25) [16]. SNP markers have been successfully applied to identify genetic determinants of growth, disease resistance, and environmental stress tolerance in various crustacean species [17,18,19]. For instance, the C14895C>T locus in the CCT6 gene was associated with growth performance in *P. vannamei*, and the PmE74-In1-53 SNP was significantly associated with low-salinity tolerance in *Penaeus monodon*, which could provide valuable markers for breeding programs. In *Eriocheir sinensis*, two SNP loci (A3088T and C2466C) in the *MIH* gene were identified as potential markers for reducing precocious maturation. In recent years, SNP detection technologies have evolved from simple, single-method approaches to high-throughput, multi-platform integrated strategies. Techniques such as genotyping-by-sequencing [20], next-generation sequencing, and high-density SNP panels enable rapid, cost-effective, and large-scale genotyping in aquaculture species, allowing customization of SNP numbers [21]. These methods have been widely applied in genomic selection, population genetics, and trait association studies [22].

The mud crab *S. paramamosain* is a euryhaline species of economic importance in aquaculture [23]. It exhibits complex developmental transitions, including zoeal, megalopa, juvenile, and adult stages [24]. Among these stages, juveniles exhibit high adaptability to low salinity and can rapidly make physiological adjustments to salinity changes; therefore, in practical aquaculture operations, low-salinity acclimation is typically conducted during this stage [25]. In recent years, climate change and coastal urbanization have intensified salinity fluctuations in aquaculture systems, posing significant challenges to crustacean production [26,27]. It is an urgent priority to expand aquaculture into low-salinity areas, which makes it a focal research field to develop salinity-tolerant strains. Despite previous reports of SNP discovery in *S. paramamosain* [28,29], there is little study on the identification of SNPs associated with important traits. In the present study, SNP markers in the *NKA* gene were screened and validated through association analysis between genotypes and low-salinity tolerance phenotypes. This study aims to identify SNP loci in the *NKA* gene that are associated with low-salinity adaptation in *S. paramamosain*. The findings will establish a theoretical foundation for marker-assisted breeding of low-salinity-tolerant strains.

## 2. Results

### 2.1. Analysis of Survival Rates of the Experimental Group and the Control Group

**Figure 1:**
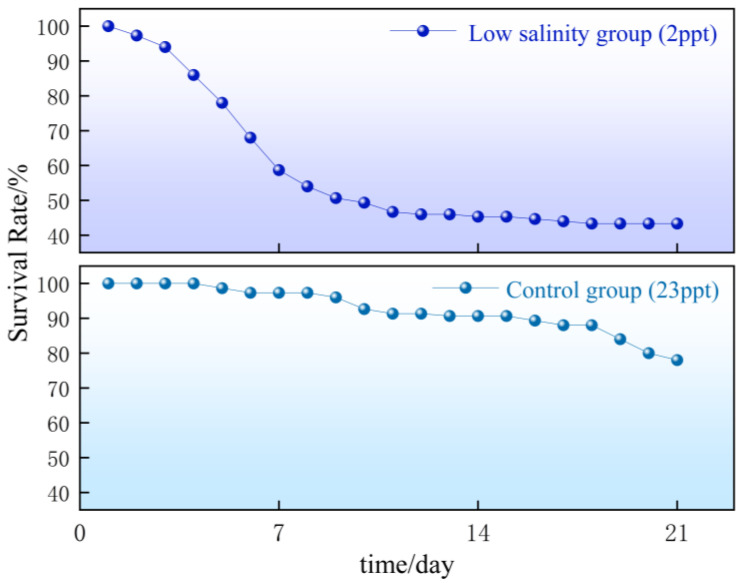
Survival rate curves of the low-salinity group and the control group. The salinity of the control group was 23 ppt, whereas that of the low-salinity group was 2 ppt.

The tolerance level of individuals was assessed by measuring the survival rates in both the control and experimental groups. The survival curves revealed a clear divergence between the groups (Figure 1), with a sharp decline in the experimental group during the first seven days, followed by stabilization, while the survival rate of the control group was relatively stable. Following the phenotypic assessment, individuals were assigned to tolerant and intolerant groups for SNP genotyping and association analysis.

### 2.2. Gene Structure and Candidate SNPs of NKA

**Figure 2:**
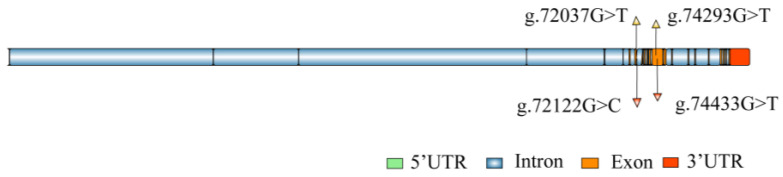
Structure of the *NKA* gene and the positions of the mutation sites. The g.72037G>T, g.72122G>C, g.74293G>T, and g.74433G>T represent four mutation sites located within the *NKA* gene.

Based on the genome data of *S. paramamosain* in the NCBI database, the structure of the *NKA* gene was constructed (Figure 2). The gene consists of 21 introns and 22 exons. A total of 15 SNPs were preliminarily screened from the *NKA* gene, with 11 located in exons and 4 in introns. Based on MAF, four loci were selected for genotyping. According to the positions of the loci on the gene, they were designated as g.72037G>T, g.72122G>C, g.74293G>T, and g.74433G>T, respectively. Of these loci, g.72037G>T was a nonsense mutation, g.74293G>T and g.74433G>T were missense mutations, while g.72122G>C was an intronic mutation (Table 1). The protein contains a Cation transport ATPase (P-type) domain, with the missense mutations p. Asp522Tyr (g.74293G>T) and p. Glu568Asp (g.74433G>T).

### 2.3. Association Analysis Between NKA Gene SNP Loci and Low-Salinity Tolerance

**Figure 3:**
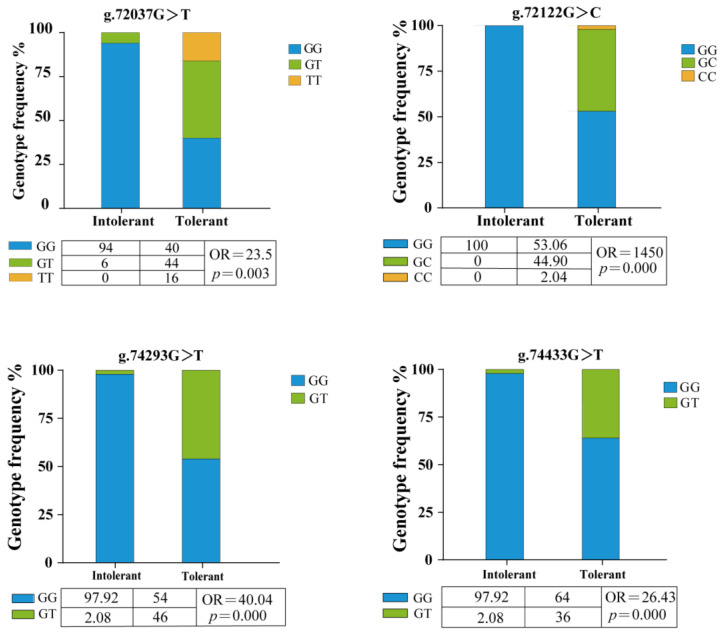
Frequencies of genotypes at four SNP loci in the *NKA* gene of *Scylla paramamosain.* A *p*-value of less than 0.05 indicates a statistically significant difference in mutant genotype frequency between the low-salinity-tolerant and low-salinity-intolerant groups.

**Figure 4:**
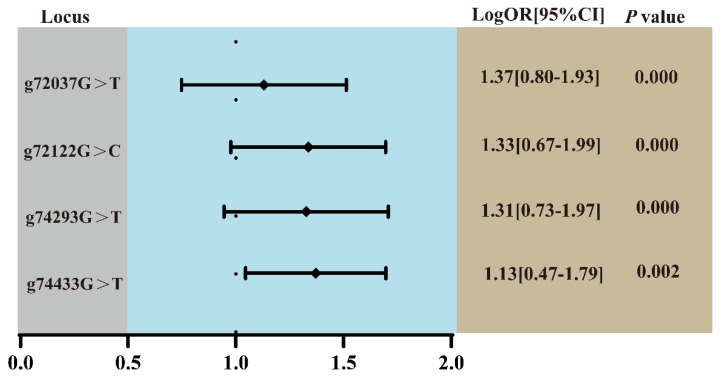
Logistic regression analysis of SNP loci associated with low-salinity tolerance phenotype. A *p*-value of less than 0.05 indicates a statistically significant difference in mutant genotype frequency between the low-salinity-tolerant and low-salinity-intolerant groups.

**Figure 5:**
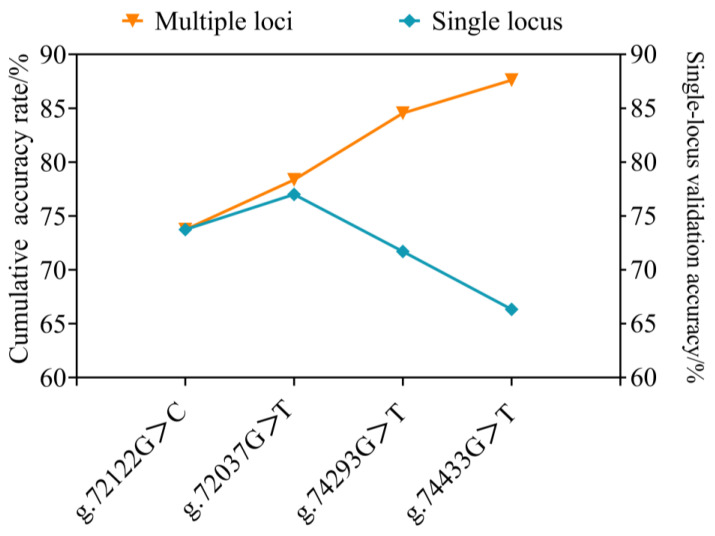
Cumulative accuracy rate of SNP loci in identifying salt-tolerance phenotypes. The blue line represents the validation accuracy of a single locus, while the orange line indicates the cumulative validation accuracy across multiple loci.

The four loci were successfully genotyped in both low-salinity-tolerant and low-salinity-intolerant groups. Both g.72037G>T and g.72122G>C exhibited three genotypes, while g.74293G>T and g.74433G>T showed only two genotypes, respectively (Figure 3). Fisher’s exact test showed significant differences in mutant genotype frequencies between tolerant and intolerant groups at all four loci (*p* < 0.05). Odds ratio analysis showed that all four SNPs had OR values more than 1. Further logistic regression analysis indicated that the mutant genotypes at all four loci significantly increased the probability of individuals exhibiting the tolerant phenotype (Figure 4). To assess genetic diversity at mutation sites, polymorphism analysis was performed between the tolerant and intolerant groups (Table 2). The tolerant group exhibited higher observed heterozygosity (Ho), expected heterozygosity (He), effective number of alleles (Ne), and polymorphic information content (PIC) at all loci than the intolerant group. This study also analyzed the frequency distribution of haplotypes between the two groups. The GGGG haplotype was predominantly associated with the intolerant group, whereas the other haplotypes were mainly enriched in the tolerant group (Table 3). The maximum accuracy was 77% when only one locus was used, while it increased to 87.62% when all four loci were jointly applied for validation (Figure 5).

### 2.4. Analysis of NKA Expression in Different Genotypes

**Figure 6:**
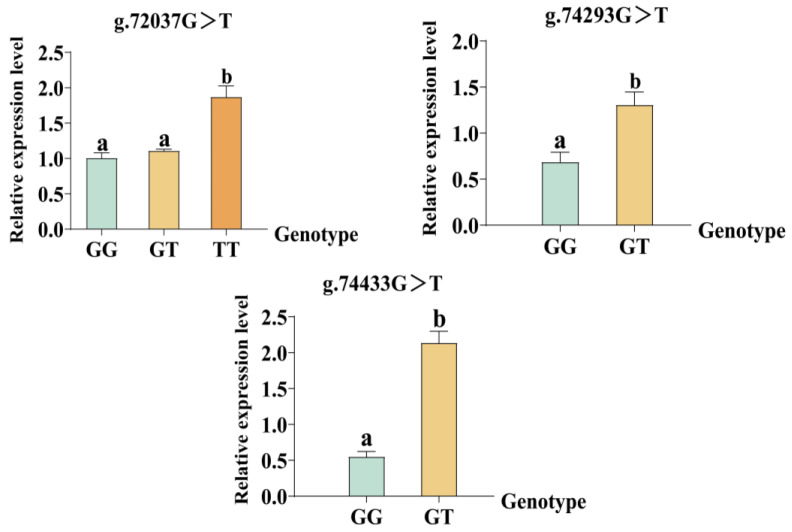
The relative expression levels of individuals with different genotypes at the mutation sites in *Scylla paramamosain.* Each genotype was measured with three biological replicates. Data are presented as mean ± SE. Statistical differences among genotypes at each locus were evaluated using one-way ANOVA followed by Tukey’s multiple comparison test. Different letters indicate significant differences (*p* < 0.05).

The expression of *NKA* showed significant differences among the genotypes in low-salinity-tolerant individuals. At the g.72037G>T locus, there was no significant difference between GG wild and GT mutant genotypes, while significant differences existed between GG wild and TT mutant genotypes (*p* < 0.05). At the g.74293G>T and g.74433G>T loci, the NKA expression in the GT mutant genotypes was significantly higher than that in the GG wild genotypes (Figure 6).

### 2.5. Association Analysis Between SNP Loci and Growth Performance

**Figure 7:**
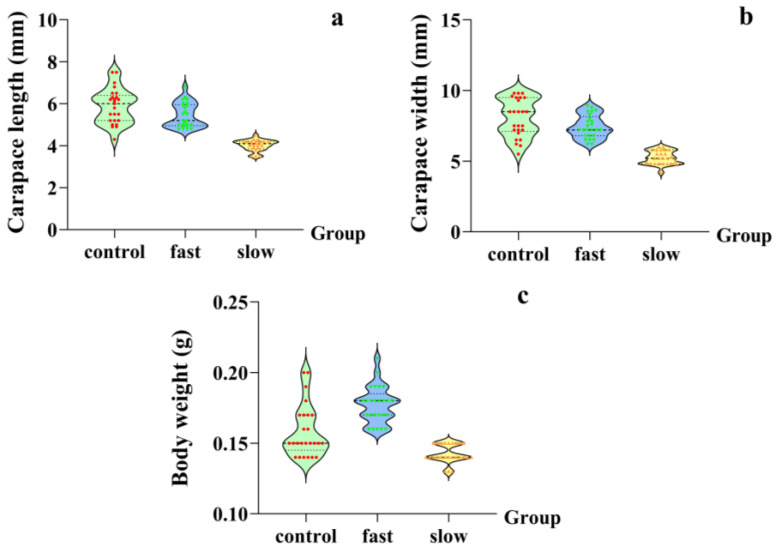
Comparison of growth performance among the fast-growing, slow-growing, and control groups: (**a**) carapace length; (**b**) carapace width; and (**c**) body weight.

The result of PCA revealed that the first two components collectively accounted for 99.60% of the total variance. PC1, with an eigenvalue of 2.890, explained 96.34% of the variance and exhibited high loadings (0.974–0.996) across all three morphological parameters, primarily reflecting overall body size. PC2, with an eigenvalue of 0.098, contributed only 3.26% of the variance (Table 4). Based on PC1 scores, growth performance among different genotypes was comprehensively evaluated. Fifty low-salinity-tolerant individuals were classified into fast-growing (*n* = 25) and slow-growing (*n* = 25) groups. Growth trait measurements for the three groups are presented in Figure 7. Overall, the fast-growing and control groups exhibited larger carapace length, carapace width, and body weight compared with the slow-growing group. Significant differences in mutant genotype frequency distribution were observed among the four SNPs between fast-growing and slow-growing groups (Table 5). Specifically, the frequencies of GT and TT mutant genotypes at the g.72037G>T locus were higher in the fast-growing group than in the slow-growing group. Similarly, the GT mutant genotypes at g.72122G>C, g.74293G>T, and g.74433G>T loci were also more prevalent in the fast-growing group.

## 3. Discussion

Na^+^/K^+^-ATPase has been identified as a candidate gene responsible for driving Na^+^ and Cl^−^ transport in the gills of crabs under low-salinity conditions [30]. In the mud crab, the Na^+^ uptake pathway involves apical Na^+^ channels functionally coupled with V-type H^+^-ATPase proton pumps and is complemented by basolateral Na^+^/K^+^-ATPase, while Cl^−^ absorption occurs through apical Na^+^-K^+^-2Cl^−^ cotransporters, contributing to osmoregulatory responses under varying salinity conditions [31,32]. Currently, most studies on *NKA* in crustaceans have focused on gene expression at the transcriptomic level, whereas research on the effects of its genetic variation on function remains limited. SNPs are the most abundant form of genetic variation in genomes. They can influence phenotypic traits through diverse molecular mechanisms depending on their genomic location and function [33,34]. In the present study, three types of SNPs were identified in the *NKA* gene: two missense mutations (g.74293G>T and g.74433G>T), one nonsense mutation (g.72037G>T), and one intronic mutation (g.72122G>C). The two missense mutations are located within the ATP hydrolysis functional domain of NKA [35,36]. Amino acid substitutions in this region might enhance catalytic efficiency and ion affinity under hypoosmotic conditions, so individuals carrying the T allele exhibited enhanced low-salinity tolerance. Although intronic variants do not alter the amino acid sequence, they can affect transcription rate and mRNA stability to influence phenotypic traits [37]. The g.72122G>C locus might exert regulatory effects on *NKA* expression by modulating transcriptional dynamics [38].

The nonsense mutation appears to involve a more complex mechanism. In this study, the g.72037G>T nonsense mutation results in a premature termination codon at the 180th amino acid. This mutation occurs after the N-terminal cation transporter/ATPase functional domain but before the E1–E2 ATPase core catalytic domain. Consequently, the mutant protein is predicted to lack the core catalytic domain responsible for ATP binding and hydrolysis. Biologically, this truncation is likely to abolish ATP hydrolysis and active ion transport functions. However, qRT-PCR revealed that the TT homozygous mutants were significantly higher than those of GG wild-type (*p* < 0.05), while GT heterozygotes showed no significant difference. This unexpected upregulation likely reflected a genetic compensation response, which may not be limited to transcriptional upregulation of the mutated gene itself, but could also involve coordinated upregulation of other ion transport genes with similar functions to *NKA* [39,40]. Single-gene loss of function can be partially or fully compensated by the enhanced activity of other functionally related members within the *NKA* regulatory network [10]. In addition, the research also found that SNP–trait associations represent statistical correlations rather than direct causal effects. This is primarily due to linkage disequilibrium (LD), in which the target SNP is highly correlated with the true causal variant in the genome. As a result, the phenotypic effect of the causal variant is “tagged” by the target SNP rather than directly measured [41,42]. Therefore, the g.72037G>T itself is unlikely to be the causal variant conferring salinity tolerance; rather, it is in strong LD with other functional variants and likely reflects the combined effects of multiple linked loci, indirectly contributing to the observed phenotypic differences. This hypothesis underscores the distinction between association signals and causal variants, but the true causal locus remains to be identified through fine-mapping and functional validation in future research. Moreover, for missense mutations, they may also influence gene expression by modifying enhancer activity or other transcriptional regulatory elements [43,44]. In our study, the tolerant genotypes might achieve Na^+^ exclusion and K^+^ retention through precise regulation of *NKA* gene spatiotemporal expression, resulting in expression activation, while wild genotypes fail to maintain ionic homeostasis due to defective gene expression regulation. The differences in expression between the two genotypes are directly transformed into ionic concentration differences [45]. These multilayered regulatory mechanisms jointly regulate the tolerance phenotype in *S. paramamosain*.

Population genetic analysis provided strong evidence for an association between mutations and low-salinity tolerance. The frequencies of mutant genotypes at the four loci, g.72037G>T, g.72122G>C, g.74293G>T, and g.74433G>T, were significantly higher in the tolerant group compared to the intolerant group, with OR exceeded one for all loci, indicating a significant positive correlation with the tolerance phenotype [46,47], logistic regression further demonstrated a substantial effect size of these mutations on the tolerant phenotype. Genetic diversity was higher in the tolerant group than in the intolerant group, potentially reflecting adaptive responses to distinct environmental pressures [48]. Haplotype analysis according to multiple SNP loci can provide richer genetic information than individual SNPs and identify the associations between candidate genes and phenotypic traits [49,50,51]. In this study, there was a significant difference between groups. The GGGG haplotype dominated in the intolerant group; conversely, GGTT and TGGG showed significantly elevated frequencies in the tolerant group. These findings demonstrate that four SNPs are strongly associated with tolerance traits by multi-locus combinations analysis. Moreover, multiple SNPs used for haplotype analysis are necessary to improve the efficacy and robustness of association [52,53]. These specific haplotypes can also serve as effective genetic markers for tolerance traits in *S. paramamosain* [54], which could provide a scientific basis for molecular marker-assisted selection breeding. Furthermore, joint analysis of loci could achieve the discrimination accuracy, indicating synergistic effects of multiple loci on low-salinity tolerance.

The SNP loci in this study were also associated with growth performance, showing fast-growing individuals carrying higher mutation frequencies than slow-growing individuals. The pleiotropic effects are common in biological systems, where functional variations can produce cascading effects on multiple traits [55]. Osmotic stress typically reduces feeding, assimilation, and growth rates in decapods [56,57]. Euryhaline decapods rely on efficient osmoregulatory mechanisms to buffer salinity stress, with *NKA* playing central roles in ion homeostasis and energy metabolism [58,59,60]. Salinity tolerance is a complex trait influenced by *NKA* and other osmotic regulation genes [61]. The discrimination accuracy of the four SNP markers achieved 87.62% in the study, but these markers capture key variations in *NKA*, but do not cover all genetic factors. Therefore, the tolerant group likely includes individuals with various tolerance levels, which is supported by studies demonstrating considerable variation in osmoregulatory efficiency [62,63,64]. These differences in osmoregulatory efficiency might result in distinct energy allocation strategies. Although an energy trade-off generally exists between osmoregulation and growth, the enrichment of mutant alleles in fast-growing individuals suggests a more efficient energy allocation strategy [65]. We proposed that these mutations in *S. paramamosain* might improve NKA catalytic efficiency, reducing ATP consumption of ion transport and allocating more energy for growth. This interpretation is consistent with the result of the correlation between mutation frequency and growth rate. The finding provides a novel insight into the adaptive evolution of euryhaline crustaceans and offers a theoretical basis for molecular breeding of low-salinity-tolerant strains in *S. paramamosain*. However, the present study is limited by a relatively small sample size, and the four SNP markers capture only partial genetic variation in *NKA*, which may not fully represent individual salinity tolerance. Future studies involving larger sample sizes and a broader range of osmotic regulation genes are warranted to further validate these markers and optimize their application in breeding low-salinity-tolerant strains.

## 4. Materials and Methods

### 4.1. Experimental Materials

For SNP screening, 32 samples were collected from four wild populations: Sanmen (Zhejiang), Fuzhou (Fujian), Shenzhen (Guangdong), and Nansha (Guangdong), with eight individuals from each population. Muscle tissue from the fourth walking leg was excised and preserved in absolute ethanol at −20 °C for DNA extraction.

The samples of juvenile I were cultured in the Ninghai Research Center of the East China Sea Fisheries Research Institute, Chinese Academy of Fishery Sciences; all samples were derived from the same biological parents. A total of nine hundred healthy, size-uniform individuals were selected and randomly distributed into control and experimental groups in a triplicate design, with each replicate containing 150 individuals, resulting in 450 individuals per group. Prior to the salinity challenge experiment, all juveniles were acclimated in an environment of 23 ppt and 26 ± 1 °C for 3 days. Record the survival data for both the experimental and control groups, and construct the survival rate curve. In the experimental group, salinity was reduced by 1 ppt every 8 h. Individuals that died during the gradual salinity reduction experiment were classified into the low-salinity intolerant group, indicating an inability to withstand progressive hypoosmotic stress and reflecting limited osmoregulatory capacity and reduced euryhaline adaptation potential. They were immediately removed and preserved in absolute ethanol. Individuals that survived both the gradual salinity reduction process and the subsequent 14-day culture period at 2 ppt, while completing at least one molt after salinity stabilization, were classified into the low-salinity tolerance group. At the end of the culture period, growth data (carapace length, carapace width, and body weight) were recorded for the final surviving individuals. These individuals were preserved in liquid nitrogen. Then, samples were used for genotyping.

### 4.2. DNA and RNA Extraction

Fifty individuals were randomly selected from each of the low-salinity-tolerant and intolerant groups for genomic DNA extraction and genotyping analysis. Genomic DNA was extracted using the TransGen Biotech Marine Animal Tissue Genomic DNA Extraction Kit (TransGen Biotech Co., Ltd., Beijing, China). DNA integrity was assessed by 1% agarose gel electrophoresis at 160 V for 25 min. Approximately 5 μL of each DNA sample was loaded per well and visualized with GeneRed. DNA was considered intact when a single high-molecular-weight band was observed without significant smearing or degradation. A 2K DNA marker was used as a size reference. The purity of DNA and RNA was determined using a Nanodrop OneC micro-spectrophotometer (Thermo Fisher, Waltham, MA, USA). Quality-validated samples were used for subsequent SNP screening experiments. Total RNA was extracted using the RNA Extraction Kit (TransGen Biotech Co., Ltd., Beijing, China) from the same 50 tolerant individuals for quantitative real-time PCR (qRT-PCR) analysis. Before use, 1% agarose gel electrophoresis was used to examine the integrity of the samples, and a NanoDrop 2000 (Thermo Fisher, Waltham, MA, USA) was used to assess the concentration and purity. cDNA was synthesized from fluorescence quantification samples according to the instructions of the TransScript^®^ One-Step gDNA Removal and cDNA Synthesis SuperMix (TransGen Biotech Co., Ltd., Beijing, China). Due to the small size of the collected juvenile crabs, tissue isolation was unfeasible. Consequently, both DNA and RNA extraction were conducted by homogenizing the entire individual.

### 4.3. SNP Screening and Genotyping

The genomic sequence of the *S. paramamosain NKA* gene was obtained from the NCBI database (GenBank accession number: NC_087179). The structural diagram of the *NKA* gene was constructed using the online software IBS2.0 [66]. Primers were designed in the segments using Primer Premier 6 software. The PCR reaction system had a total volume of 25 μL, containing 9.5 μL ddH_2_O, 12.5 μL PCR mix, 1 μL DNA template, and 1 μL of each primer. The amplification program was as follows: 94 °C for 5 min, followed by 35 cycles of denaturation at 94 °C for 40 s, 56 °C for 40 s, and 72 °C for 40 s, with final extension at 72 °C for 5 min. After verification of amplification products by agarose gel electrophoresis, qualified products were sent for sequencing. Sequencing data were first trimmed to remove primer sequences and low-quality bases (Phred score < 30) at both ends, then aligned with the reference genomic sequence of the *NKA* gene using Sequencher 5.4 software to identify potential SNPs. If excessive trimming of the sequences results in reduced alignment coverage, 3–5 bases may be restored at the sequence ends before further alignment with the reference gene sequence. Data analysis was performed on potential SNPs to obtain candidate SNPs. The height of the secondary peak corresponding to the heterozygous allele must be at least 30% of the height of the primary peak. Peaks with heights less than 20% are considered noise [67]. Candidate SNPs were further confirmed by cross-checking multiple samples and filtering loci with minor allele frequency (MAF) ≥ 5% [68] (for 32 individuals, 64 alleles, this corresponds to at least four minor alleles). Those loci satisfying the criterion were selected from the candidate sites for genotyping using direct sequencing. The primer sequences used for screening and genotyping are shown in Table 6.

### 4.4. Association Between SNP Genotypes and NKA Gene Expression Levels

Primers were designed in the CDS region of the *NKA* gene using Primer Premier software. Since 18SrRNA (GenBank accession number KC902763.1) is stably expressed in different tissues of *S. paramamosain* [69], it was used as a reference gene in this experiment. The primer sequences are shown in Table 5. cDNA was synthesized using a reverse transcription kit (TransGen Biotech Co., Ltd., Beijing, China), with the thermal protocol comprising incubation at 42 °C for 15 min followed by a denaturation step at 85 °C for 5 s. Based on the genotyping results, three individuals per genotype at each locus were randomly selected for qRT-PCR analysis, and three technical replicates were performed for each individual. It was performed in a 7900 Genetic Analyzer System (Applied Biosystems, Foster City, CA, USA), and the results were analyzed by the 2^−ΔΔCt^ method [70] to evaluate the differences in NKA gene expression among genotypes under low-salinity conditions.

### 4.5. Statistical Analysis

Sequencing results were analyzed using Chromas to identify genotypes according to base types at target SNP loci [71,72]. The odds ratio (OR) for mutant genotypes versus wild types was calculated according to a codominant model [43] using the formula:

(1) $$OR=\frac{N_{1}\times N_{4}}{N_{2}\times N_{3}}$$

where N_1_ and N_2_ represent the number of mutant and wild-type individuals in the tolerant group, and N_3_ and N_4_ represent the number of mutant and wild-type individuals in the intolerant group, respectively.

Genetic parameters, including heterozygosity and polymorphic information content, were calculated using PowerMarker V3.25 [73]. Fisher’s exact test was used to preliminarily evaluate the association between genotypes and low-salinity tolerance. To further quantify the contribution of mutations at different loci to low-salinity tolerance probability, a binary logistic regression model was applied to measure the strength of change in tolerance probability induced by mutant genotypes relative to reference genotypes. Due to the proportions of some SNP genotypes differing markedly between tolerant and intolerant groups, using OR directly may be biased or unstable. Therefore, log-OR was used as the effect size and for visualization [74,75]. The haplotype frequencies were calculated using SHEsis (http://shesisplus.bio-x.cn/SHEsis.html, accessed on 20 September 2025) [76], and differences in haplotype frequencies between tolerant and intolerant groups were assessed at a significance level of *p* < 0.05. Protein domain analysis was conducted using HMMER (https://www.ebi.ac.uk/Tools/hmmer/search/phmmer, accessed on 20 September 2025). A total of 56 low-salinity-tolerant individuals were used for growth analysis. To investigate the differences in growth traits among groups, principal component analysis (PCA) was performed using carapace length, carapace width, and body weight as variables [77]. Based on the PCA scores, the 25 individuals with the highest scores were assigned to the fast-growing group, and the 25 individuals with the lowest scores were assigned to the slow-growing group. Quantitative fluorescence results were analyzed by one-way analysis of variance (ANOVA), followed by Duncan’s test when significant differences were detected. All data are expressed as mean ± standard error (SE).

For multi-locus discrimination analysis, genotype frequencies at each SNP locus were used to estimate the probability of individuals belonging to tolerant or intolerant phenotypes. A sample was defined as tolerant if its genotype frequency was higher than that in the intolerant group. Conversely, it was classified as intolerant if the frequency was lower. For multi-locus combinations, genotype frequencies across loci were multiplied to obtain two composite probability scores, and the higher score determined the phenotype classification. The validation accuracy (R) was calculated as

(2) $$R=\frac{N_{correct}}{N_{all}}\times 100\%$$

where N_correct_ is the number of correctly judged samples, and N_all_ is the total number of samples [78].

## 5. Conclusions

This study identified four SNPs (g.72037G>T, g.72122G>C, g.74293G>T, and g.74433G>T) in the *NKA* gene that are significantly associated with low-salinity tolerance in *S. paramamosain*. Mutant genotypes at these sites enhanced low-salinity tolerance and exhibited pleiotropic effects on both tolerance and growth performance, revealing the coordinated roles of *NKA* in osmoregulation and energy metabolism. These validated markers could provide effective tools for marker-assisted breeding of low-salinity-tolerant *S. paramamosain* strains. Although the four SNPs account for only part of the genetic variation in salinity tolerance, incorporating markers from other osmoregulatory genes could further enhance marker-assisted selection efficiency. Future studies should incorporate more candidate genes and systematically investigate gene–environment interactions to comprehensively elucidate the molecular mechanisms underlying low-salinity adaptation, thereby improving the efficiency and reliability of marker-assisted selection.

## Figures and Tables

**Table 1:** Summary of four SNPs identified in the *NKA* gene.

Locus	Allele	Amino Acid	Mutation Type	Domain Location	Position
g.72037G>T	G-T	E-END	Nonsense mutation	/	Exon
g.72122G>C	G-C	/	/		Intron
g.74293G>T	G-T	D-Y	Missense mutation	Cation transport ATPase (P-type)	Exon
g.74433G>T	G-T	E-D	Missense mutation	Cation transport ATPase (P-type)	Exon

Note: E, D, and Y represent glutamic acid, aspartic acid, and tyrosine, respectively. END represents a stop codon.

**Table 2:** Statistical analysis of genetic parameters at four SNPs in the *NKA* gene between different groups.

Group	Locus	Ho	He	Ne	PIC
Intolerant group	g.72037G>T	0.0300	0.0582	1.0617	0.0565
	g.72122 G>C	0.0000	0.0000	1.0000	0.0000
	g.74293G>T	0.0200	0.0205	1.0210	0.0203
	g.74433G>T	0.0200	0.0205	1.0210	0.0203
Tolerant group	g.72037G>T	0.4400	0.4712	1.8910	0.3602
	g.72122 G>C	0.4490	0.3698	1.5869	0.3015
	g.74293G>T	0.4600	0.3542	1.5484	0.2915
	g.74433G>T	0.3600	0.2952	1.4188	0.2516

Note: Ho represents observed heterozygosity, He represents expected heterozygosity, Ne represents the number of effective alleles, and PIC represents polymorphic information content.

**Table 3:** Distribution and association analysis of haplotypes from four SNPs between tolerant and intolerant groups.

Haplotype	Tolerant Group (Freq.)	Intolerant Group (Freq.)	Chi2	Fisher’s *p*	Pearson’s *p*	Odds Ratio [95%CI]
GGGG	45.53 (0.465)	93.00 (0.969)	59.251	2.08 × 10^−14^	1.45 × 10^−14^	0.029 [0.008~0.097]
GGTT	9.49 (0.097)	1.00 (0.010)	7.182	0.0074	0.0074	10.314 [1.287~82.643]
TGGG	13.00 (0.133)	2.00 (0.021)	8.642	0.0033	0.0033	7.286 [1.597~33.234]
GGTG	5.98 (0.061)	0.00 (0.000)	6.122	0.0134	0.0134	-
TCGG	15.41 (0.157)	0.00 (0.000)	16.595	4.70 × 10^−5^	4.68 × 10^−5^	-
TCGT	1.06 (0.011)	0.00 (0.000)	-	-	-	-
TCTG	0.08 (0.001)	0.00 (0.000)	-	-	-	-
TCTT	7.45 (0.076)	0.00 (0.000)	7.681	0.0056	0.0056	-

Note: Haplotype: Haplotypes constructed from four SNP loci of the *NKA* gene; Tolerant group (freq.): Absolute frequency (relative frequency in parentheses) of each haplotype in the low-salinity tolerant group; Intolerant group (freq.): Absolute frequency (relative frequency in parentheses) of each haplotype in the low-salinity intolerant group; Chi2: Chi-square test statistic; Fisher’s *p*: *p*-value of Fisher’s exact test; Pearson’s *p*: *p*-value of Pearson’s chi-square test; Odds Ratio [95%CI]: Odds ratio and its corresponding 95% confidence interval; “-“: Indicates that the statistical index cannot be calculated, caused by zero frequency of the haplotype in the corresponding group.

**Table 4:** Principal component analysis of growth traits in the low-salinity-tolerant group.

Component Loadings	PC1	PC2
Carapace width (mm)	0.974	0.224
Carapace length (mm)	0.975	−0.218
Body weight (g)	0.996	−0.006
Eigenvalue	2.89	0.098
Variance explained (%)	96.341	3.26
Cumulative variance (%)	96.341	99.6

Notes: Component loadings: Loading values of each growth trait (carapace width, carapace length, body weight) on Principal Component 1 (PC1) and Principal Component 2 (PC2); Carapace width (mm), Carapace length (mm), Body weight (g): Growth trait indicators and their corresponding units; Eigenvalue: Eigenvalue of each principal component; Variance explained (%): Proportion of total variance explained by a single principal component; Cumulative variance (%): Cumulative proportion of total variance explained by the principal components.

**Table 5:** The differences in the mutant genotype frequency distribution of the four SNPs between the fast-growing group and the slow-growing group.

Locus	Fast-Growing Group		Slow-Growing Group	
	Number	Frequency (%)	Number	Frequency (%)
g.72037G>T	21	0.7	9	0.3
g.72122G>C	19	0.79	4	0.21
g.74293G>T	14	0.58	9	0.42
g.74433G>T	12	0.67	6	0.33

**Table 6:** PCR primers and corresponding sequences used in this study.

Primer Name	Sequence (5′~3′)	Purpose	Product Size
Sp-NKA-F1	CTCGCTGACACCTAGGCCC	SNP screening	420 bp
Sp-NAK-R1	TTTCCAAGCACAACCATTAGCTG		
Sp-NKA-F2	TACTTGGGCATTGTGCTCAC	SNP screening	362 bp
Sp-NKA-R2	GAAACCTCGAGCTTCAATGACT		
Sp-NKA-F3	GTATGCCATTGTCATCCGTGAG	SNP screening	453 bp
Sp-NKA-R3	CCTTCAACAGCATTGGTGGAA		
Sp-NKA-F4	TTGGTGACAATACTGTGATGGG	SNP screening	607 bp
Sp-NKA-R4	AGCCTGACTGGTCCTCTGA		
Sp-NKA-F5	TGCCAGTATGACAAGACCTCTG	SNP screening	564 bp
Sp-NKA-R5	ACGGGGAGGATCAATCATGG		
Sp-NKA-F6	CATTGCCAAGTCTGTCGGTAT	SNP screening	691 bp
Sp-NKA-R6	ACCATGTCAGTACCCAGATCA		
Sp-NKA-F7	GTTTGGTGGATTCTCACTGCTG	SNP genotyping	171 bp
Sp-NKA-R7	AACCATTAGCTGAGGCAGTAACTAT		
Sp-NKA-F8	ATCAGAGGAGGAACCCAACAAT	SNP genotyping	179 bp
Sp-NKA-R8	TCCCTTCCTCTAACAGAACCCC		
Sp-NKA-F9	CTGAAGCGAGAGGTAAACGGT	SNP genotyping	159 bp
Sp-NKA-R9	GGTCTCATGGATGGACACTTGA		
Sp-NKA-F10	AGATTCCTTTCAACTCCACCAACA	SNP genotyping	180 bp
Sp-NKA-R10	TGAAAGCTTCCTTCATCTCCTCAT		
Sp-NKA-RT-F	ATGGCATGGACAGCCATCTC	qRT-PCR	121 bp
Sp-NKA-RT-R	CTATCCCCTGTTGTCGCTGG		
Sp-18S-RT-F	GGGGTTTGCAATTGTCTCCC	qRT-PCR	92 bp
Sp-18S-RT-R	GGTGTGTACAAAGGGCAGGG		

## Data Availability

The raw data supporting the conclusions of this article will be made available by the authors on request.
